# Serum Metabolomics of Patients with Hepatic Cystic Echinococcosis

**DOI:** 10.1002/bmc.70180

**Published:** 2025-09-08

**Authors:** Yisimayili Aimaiti, Kahaer Tuerxun, Yuan‐Quan Wu, Abudoukeyimu Yasheng, Irshat Ibrahim, Qi‐Lin Xu, Muzaipaer Muhetajiang, Tuerganaili Aji

**Affiliations:** ^1^ Department of Hepatobiliary Hydatid Surgery The First Affiliated Hospital of Xinjiang Medical University Urumqi Xinjiang Uygur Autonomous Region China; ^2^ Department of Hepatobiliary Surgery The First People's Hospital of Kashi Prefecture Kashi China

**Keywords:** hepatic cystic echinococcosis, metabolomics, serum, untargeted metabolomics experiment

## Abstract

Hepatic cystic echinococcosis (HCE), a liver manifestation of hydatid disease, is among the 17 neglected tropical diseases (NTDs) prioritized by the WHO for eradication by 2025. Although imaging and serological tests are currently the main diagnostic approaches for HCE, they have notable limitations in sensitivity and specificity. Here, we applied liquid chromatography–tandem mass spectrometry (LC‐MS/MS)–based metabolomic profiling to uncover differential metabolites and highlight disrupted metabolic pathways, aiming to identify candidate biomarkers for HCE diagnosis. Ten patients diagnosed with HCE were enrolled in the case group. Thirteen healthy individuals were included as controls. Serum metabolomic profiling was performed using LC‐MS/MS. Differences in metabolite profiles between the two groups were analyzed employing both univariate and multivariate statistical methods. A total of 20 differential metabolites were significantly altered in the HCE group compared to the controls (*p* < 0.05, VIP > 1.0). Pathway and enrichment analyses revealed that these metabolites were mainly involved in 8 metabolic pathways, suggesting their potential as candidate biomarkers for HCE diagnosis. These findings enhance our understanding of the metabolic alterations associated with HCE and provide a foundation for further investigation into the disease's pathogenesis and the development of metabolite‐based diagnostic tools.

## 1 Introduction

Echinococcosis is a serious zoonotic infection caused by parasitic tapeworms of the genus *Echinococcus* (Zhang and Zhou 2021), with hepatic cystic echinococcosis (HCE) listed among 17 neglected tropical diseases (NTDs) prioritized by the World Health Organization (WHO) for elimination by 2025 (Wen et al. 2019). Human hepatic echinococcosis primarily manifests in two forms: cystic echinococcosis, caused by *Echinococcus granulosus*, and alveolar echinococcosis, caused by *Echinococcus multilocularis*. The liver is most frequently involved, although secondary involvement may occur in the lungs, brain, bones, kidneys, and other tissues (Bhutani and Kajal 2018; Govindasamy et al. 2023). HCE‐associated mortality is lower, typically 2%–4% (Wen et al. 2019).

In recent years, the global burden of echinococcosis has escalated into a significant public health concern (Yang et al. 2024). Contributing factors include the expansion of the tourism industry, increased human mobility, and a rapid rise in the dog population (Tian et al. 2024). In endemic regions, the annual incidence of HCE varies widely, from 1 to 200 cases per 100,000 individuals (“Chinese Expert Consensus on Diagnosis and Treatment of Hepatic Cystic and Alveolar Echinococcosis (2019 Edition),” 2019). In China's western regions, the average prevalence is approximately 1.08%, with some areas on the Qinghai‐Tibet Plateau reporting rates as high as 6%. An estimated 60 million people are at risk, resulting in annual direct economic losses of up to 3 billion yuan (Wen and Xu 2007).

Diagnosis of HCE primarily relies on imaging techniques, as most patients remain asymptomatic during the early stages, posing significant challenges to timely diagnosis. Moreover, the cysts associated with HCE typically grow very slowly (Kern et al. 2017).

Although imaging and serological tests remain the primary methods for diagnosing HCE, both approaches have notable limitations. Imaging techniques often lack the sensitivity to detect early‐stage lesions smaller than 1 cm, while serological tests are prone to cross‐reactivity, particularly with antibodies generated in response to other parasitic infections (Zhao and Shen 2023). Metabolomics offers a novel and complementary diagnostic approach by capturing small‐molecule metabolic changes that occur during host–parasite interactions: (1) Metabolite changes typically precede anatomical changes. For example, in Alzheimer's disease, metabolite‐based biomarkers have been shown to predict onset 5–10 years before any structural changes are detectable via imaging (Oka et al. 2024); (2) specific disruptions in host metabolic pathways can reflect the parasite's ability to hijack hepatic metabolism—information that imaging alone cannot provide; (3) metabolomic analysis has demonstrated diagnostic potential in echinococcosis and can enhance diagnostic accuracy when used alongside conventional imaging techniques (Li and Hou 2022).

In the early phase, clinical symptoms are often absent, and over half of the cysts may remain unchanged in size for 10–15 years (Romig et al. 2006). However, rupture of the cysts can lead to severe complications. Leakage of cyst fluid into the peritoneal cavity—rich in antigenic components—can provoke anaphylactic shock and intense allergic reactions. Additionally, cyst rupture may result in intra‐abdominal infections, manifesting as peritonitis with symptoms like abdominal pain, guarding, and fever (Gundappa et al. 2022). Communication between cysts and the biliary tract can cause cholangitis, characterized by chills, high fever, and jaundice (Suleimanov et al. 2023).

Recent advances have highlighted the potential of metabolomics in the early diagnosis and therapeutic monitoring of diseases. Metabolomics reflects the dynamic biological responses of cells during pathophysiological processes and captures metabolic alterations induced by both intracellular and extracellular stimuli (Deplazes et al. 2017). Nontargeted metabolomics studies on patients with echinococcosis have shown that the disease induces significant disturbances in fundamental metabolic processes, resulting in distinct metabolic profiles compared to healthy individuals (Fernie 2007; Reaves and Rabinowitz 2011). By comparing specific metabolites between diseased and healthy individuals, potential diagnostic biomarkers can be identified. Typically, high‐throughput techniques are employed to analyze biological samples such as blood or urine, producing spectral data that is subsequently processed using statistical software to extract meaningful results (Laíns et al. 2019). Unlike genomics, transcriptomics, or proteomics, which focus on specific genes or proteins, metabolomics investigates a wide range of metabolic compounds, which involves fewer targets, employs more accessible methodologies, and is particularly sensitive to subtle changes in gene and protein expression (Ghatak et al. 2018; Smith et al. 2006). Furthermore, metabolomics provides a valuable link between endogenous and exogenous metabolite levels in cells and body fluids and overall physiological function, offering a powerful tool to elucidate host–parasite interactions (Mihmanli et al. 2016; Nicholson et al. 2012).

Metabolites thus hold promise as potential biomarkers for the early detection of HCE. This study probes into the pathogenesis of HCE and aims to establish effective diagnostic strategies through the identification of disease‐specific markers using metabolomics (Raj et al. 2024). By analyzing serum metabolic profiles in HCE patients and healthy controls, we aim to provide a molecular foundation for understanding host–parasite interactions in the context of HCE.

## 2 Materials and Methods

### 2.1 Research Subjects

This case–control study was conducted at the First People's Hospital of Kashgar Region, Xinjiang, China, in which 10 patients who had been diagnosed with HCE between January 2023 and December 2024, based on both imaging findings and serological tests, were enrolled as the case group. To establish a control group, 13 healthy individuals, who had been matched for age and sex with the case group and who underwent routine examinations at the hospital's health check‐up center, were selected. The overall cohort, in which the male‐to‐female ratio was 5.5:6, included participants whose mean ages were 36.2 ± 12.4 years for the case group and 37.9 ± 8.1 years for the control group. Six male patients and four female patients; nine patients underwent cystectomy and one underwent hepatectomy, with no postoperative complications (Table 1).

**TABLE 1:** Clinical characteristic of HCE patients.

Index	Sex	Age (years)	Location	Size (cm)	Cyst types	Complication	Type of surgery	Anthelmintic therapy
1	Male	18	VI	5 × 6	ce2	Grade1	Cystectomy	No
2	Male	28	V VIII	7 × 8	CE2	Grade1	Cystectomy	No
3	Female	39	VI‐VII	9 × 7	ce1	Grade1	Cystectomy	No
4	Male	53	II‐III	6 × 8	CE1	Grade2	Hepatectomy	No
5	Female	22	VI	5 × 7	CE1	Grade1	Cystectomy	No
6	Male	42	VII	9 × 11	ce3	Grade1	Cystectomy	No
7	Male	24	V‐VI	10 × 8	CE2	Grade1	Cystectomy	No
8	Female	32	VII	7 × 9	CE1	Grade1	Cystectomy	No
9	Male	55	VI	8 × 9	CE1	Grade1	Cystectomy	No
10	Female	47	V VI	6 × 7	CE2	Grade1	Cystectomy	No

#### 2.1.1 Inclusion Criteria (Case Group)

(1) Written informed consent was secured from each participant prior to enrollment. (2) Only newly diagnosed, primary HCE cases with positive serological (antibody) tests were included. (3) Diagnosis was confirmed by both imaging and serology. Patients had adequate cardiac and pulmonary function to undergo surgery. (4) HCE lesions were classified as unicystic, multicystic, or collapsed endocyst types (Association 2019). (5) Complete medical records were available for clinical data collection. (6) Participants were between 18 and 55 years of age. (7) Control group individuals were clinically healthy, with no known medical conditions, as verified through routine health examinations.

#### 2.1.2 Exclusion Criteria (Case Group)

1. Presence of tumors, hypertension, coronary heart disease, liver cirrhosis, renal insufficiency, metabolic disorders, or hematological diseases. (2) Use of antibiotics, corticosteroids, or nonsteroidal anti‐inflammatory drugs (NSAIDs) within the previous 2 weeks. (3) Use of antiparasitic medication within the past 3 months. (4) Major physiological stress events or exposure to radiotherapy/chemotherapy within 2 weeks prior to blood sampling. (5) History of blood transfusion within the past 6 months. (6) Recurrent echinococcosis. (7) Cases with solid or calcified cyst types. (8) Presence of other space‐occupying lesions in the liver.

### 2.2 Specimen Preparation

Fasting venous blood samples (2 mL) were collected from both patients and healthy controls in the morning prior to surgery or medical intervention. The serum was separated by centrifugation within 30 min of collection and stored at −80°C until analysis.

### 2.3 Ethical review

This study was conducted in strict accordance with the Declaration of Helsinki and the ethical guidelines of the International Committee of Medical Journal Editors. The study protocol (No.: KDYY‐EC‐SOP‐009‐03.0) was reviewed and approved by the Ethics Committee of the First People's Hospital of Kashgar, Xinjiang (approval date: 2025‐04‐30). All participants signed informed consent forms before enrollment, acknowledging their awareness of the study's purpose, data usage, and privacy safeguards. Serum sample collection, storage, and use complied with the committee's standards (SOP No.: KDYY‐EC‐SOP‐009‐03.0), which ensured voluntary participants the right to withdraw at any time, anonymization of personal data, and the exclusive use of samples for metabolomics analysis within this study.

### 2.4 Serum Metabolomics Experiment

Serum (100 μL) was deproteinized with 300 μL of MeOH:ACN (2:1, v/v) at 4°C for 10 min, then centrifuged at 12,000 rpm for 10 min at 4°C. The resulting supernatant (200 μL) was transferred to labeled tubes and stored at −20°C for 30 min to precipitate residual proteins. After thawing on ice, the samples were subjected to a second centrifugation (12,000 rpm, 3 min, 4°C) to remove any additional precipitates. A final aliquot of 180 μL was loaded into autosampler vials for LC‐MS/MS analysis. A total of 23 samples (HCE patients vs. healthy controls) underwent untargeted metabolomic profiling. The chromatographic conditions were as follows: column, Waters ACQUITY Premier HSS T3 (1.8 μm, 2.1 mm × 100 mm); mobile phase A, 0.1% formic acid in water; mobile phase B, 0.1% formic acid in acetonitrile; column temperature, 40°C; flow rate, 0.4 mL/min; injection volume, 4 μL.

### 2.5 System Stability

Quality control (QC) samples were interspersed at 10‐sample intervals to assess instrument consistency and data reliability. The stability of the analytical system was evaluated based on the total ion current (TIC) chromatograms under both positive and negative ion modes. High overlap of TIC curves, consistent retention times, and peak intensities, along with clear peak separation, indicated that the system exhibited good stability and reproducibility, thus ensuring the reliability of the metabolomics data.

### 2.6 Data Extraction and Pretreatment

Raw mass spectrometry (MS) data were first converted to .mzXML format utilizing ProteoWizard software. Data processing, including peak detection, alignment, and retention time correction, was performed using the XCMS package. Peaks with missing values in more than 50% of the samples were excluded. The remaining missing values were imputed using the k‐nearest neighbors (KNN) algorithm. Signal intensities (peak areas) were normalized using support vector regression (SVR) to minimize systematic variation. Metabolite identification was conducted using both in‐house and public databases, in combination with metDNA. Only metabolites with identification scores ≥ 5 and coefficients of variation (CV) < 3% in QC samples were retained. The identification score was calculated based on multiple parameters, including MS/MS spectral library matching, retention time deviation, and isotopic pattern distribution. The threshold for identification confidence was set according to the classification criteria proposed by Schymanski et al. Data from positive and negative ion modes were merged, with the metabolite showing the smallest CV retained when duplicates were detected. This process yielded a comprehensive ALL sample data matrix for subsequent analysis.

### 2.7 Statistical Analysis

No significant differences were observed between the two groups in terms of sex, age, BMI, white blood cell count, hemoglobin levels, liver and kidney function, or fasting blood glucose (*p* > 0.05). To comprehensively explore the data and accurately identify differential metabolites, both univariate and multivariate statistical analyses were employed based on the characteristics of the dataset. Univariate analysis included hypothesis testing and fold change (FC) analysis. Multivariate statistical methods comprised principal component analysis (PCA), orthogonal partial least squares discriminant analysis (OPLS‐DA), cluster analysis, and pathway enrichment analysis. The variable importance in projection (VIP) scores derived from the OPLS‐DA model (with biological replicates ≥ 3) were used for the preliminary identification of differential metabolites between varieties or tissue types. *p* < 0.05 was deemed statistical significance.

## 3 Results

### 3.1 PCA Model

Prior to differential analysis, PCA was conducted on all samples—including QC samples—to assess both intergroup separation and intragroup variability. The PCA results revealed distinct metabolic separation trends between the groups, while also providing insights into the variability within each group.

**FIGURE 1:**
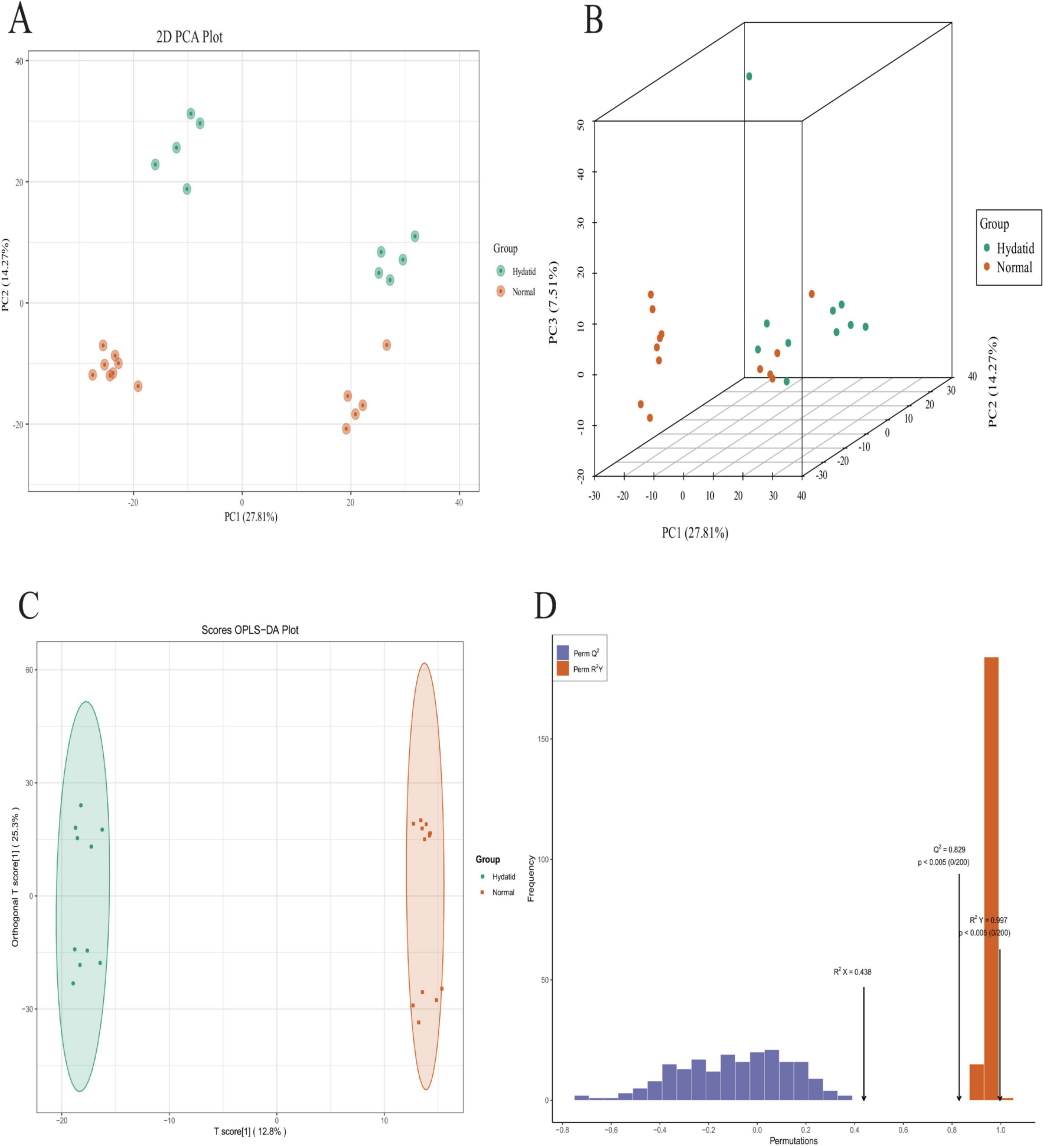
(A) PCA score plots. The percentage shown on each axis indicates the proportion of variance in the dataset explained by the corresponding principal component. Each point represents an individual sample. (B) Three‐dimensional PCA plot showing group distribution along the first three principal components (PC1, PC2, and PC3), which represent the directions of maximum variance in the data. (C) OPLS‐DA Score Plot comparing the hydatid disease group and the control group. The plot reveals both intergroup separation and intragroup variability. The percentages on the axes indicate the proportion of variance in the dataset explained by the corresponding components. (D) Validation plot of the OPLS‐DA model. The orange bars represent the *R* ^2^ *Y* values from the permutation tests, while the purple bars represent the corresponding *Q* ^2^ values. The black arrow denotes the *R* ^2^ *X*, *R* ^2^ *Y*, and *Q* ^2^ values of the original, nonpermuted model.

As shown in Figure 1A, the PCA score plot indicates a clear trend of group separation, with each point representing an individual sample. The percentages on the axes represent the proportion of variance explained by the corresponding principal components. The first three principal components accounted for 24.48%, 12.31%, and 7.52% of the total variance, respectively. A three‐dimensional PCA plot further highlights the spatial distribution of the two groups along the three main components (Figure 1B), suggesting distinct metabolomic profiles in the HCE group compared to controls.

### 3.2 OPLS‐DA Model Results

To better distinguish the metabolic profiles between groups, OPLS‐DA was applied. This supervised technique decomposes the X matrix into Y‐predictive and Y‐orthogonal components, effectively isolating variance relevant to class separation. Based on this decomposition, OPLS‐DA score plots were generated to visualize group differences and facilitate the interpretation of the underlying metabolic alterations.

While PCA offers an unsupervised overview of the data structure, OPLS‐DA enhances class separation by maximizing between‐group variance. As illustrated in Figure 1C, the OPLS‐DA score plot demonstrates clear clustering of the hydatid group and the control group, reflecting distinct metabolic profiles. Model performance was evaluated utilizing the parameters *R* ^2^ *X*, *R* ^2^ *Y*, and *Q* ^2^, which represent the model's goodness‐of‐fit and predictive ability. The model yielded a *Q* ^2^ value of 0.829, suggesting high predictive reliability and robustness. These results, further supported by permutation validation (Figure 1D), highlight the presence of significant metabolic distinctions between the experimental and control groups .

### 3.3 Distribution and Screening of Differential Metabolites

**FIGURE 2:**
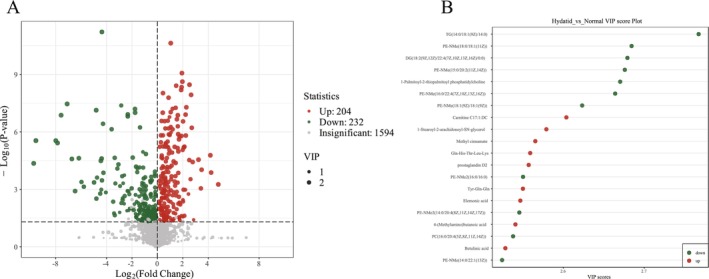
(A) Volcano plot of differential metabolites. Each point represents a metabolite: red indicates upregulated, green indicates downregulated, and grey indicates metabolites with no significant difference between groups. The *x*‐axis shows the log_2_FC in metabolite levels, where a larger absolute value indicates a greater magnitude of change. The *y*‐axis represents statistical significance (−log_10_ *p* value). Metabolites were filtered based on VIP, FC, and *p* value. The size of each dot reflects its corresponding VIP score. (B) VIP plot of differential metabolites. The *x*‐axis denotes the VIP value, while the *y*‐axis lists the names of selected differential metabolites. Red indicates upregulated metabolites, green indicates downregulated metabolites, and yellow denotes metabolites that showed significant differences in three or more comparison groups.

To visually illustrate the overall metabolic differences between two groups, FC values of metabolites were calculated. Preliminary screening of differential metabolites was conducted using the VIP scores derived from the OPLS‐DA model (biological replicates ≥ 3) (Figure 2B). Further refinement of differential metabolite selection was achieved by integrating results from univariate analysis, including *p* values, false discovery rates (FDR), and FC values (biological replicates ≥ 2).

Metabolites were considered significantly different based on two criteria: (1) VIP > 1, which typically suggests biological relevance in group separation; and (2) *p* < 0.05, indicating statistical significance in metabolite abundance between groups. A volcano plot (Figure 2A) was prepared to visually represent the distribution of differentially expressed metabolites between the case and control groups.

Here, serum metabolomic profiling of patients with HCE and healthy controls identified a total of 436 significantly altered metabolites. Under positive (POS) and negative (NEG) ionization modes, 312 and 124 differential metabolites were detected, respectively. Among these, 204 metabolites were upregulated, and 232 were downregulated (*p* < 0.05, VIP > 1), indicating marked metabolic differences between the HCE and control groups. An additional 1,594 metabolites showed no significant differences (Figure 2A).

To further refine the analysis, differential metabolites were ranked based on their FC values. The top 20 metabolites showing the most significant alterations were selected for detailed analysis.

These 20 metabolites are listed in Table 2. Among them, 12 metabolites were found to be significantly elevated in the serum of HCE patients relative to healthy individuals. These include alcohols and amines, hormones, carbohydrates, fatty acids, organic acids, amino acids and their derivatives, benzenes and derivatives, sphingolipids. Conversely, 8 metabolites were significantly reduced in HCE patients: amino acids, organic acids, alcohols, amines and derivatives, aldehydes, ketones, esters, heterocyclic compounds, and glycerophospholipids. All identified metabolites met the significance criteria of *p* < 0.05 and VIP > 1.0. These findings indicate that alterations in lipid metabolism, amino acid, fatty acid, organic acid, amine, hormone, carbohydrate, and benzene derivative are closely associated with *Echinococcus granulosus* infection in HCE patients.

**TABLE 2:** Results of differential metabolite screening between serum of HCE patients and healthy individuals.

Index	Compounds	VIP	*p*	Fold change	Variation trend
1	Allantoic acid	1.58	0.00750	0.6433810	Down
2	N‐(2‐hydroxyethyl‐1,1,2,2‐d4)‐5Z,8Z,11Z,14Z‐eicosatetraenamide	1.80	0.00020	1.2029708	Up
3	Rengyol	1.34	0.01222	0.8877841	Down
4	Prostaglandin A1 ethyl ester	1.31	0.04617	1.4076302	Up
5	D‐Erythrulose 4‐phosphate	1.47	0.01745	1.2121655	Up
6	Propyl (9E)‐12‐hydroxyoctadec‐9‐enoate	1.96	0.00023	1.5386944	Up
7	Dacarbazine	1.31	0.01406	0.8928246	Down
8	Butanoic acid, 4‐((1,2‐dioxohexadecyl)amino)‐, ethyl ester	1.86	0.00112	1.5056723	Up
9	N‐[(3S)‐Tetrahydro‐2‐oxo‐3‐furanyl]hexadecanamide	1.97	0.00029	1.5477792	Up
10	15(S)‐HETE‐d8	1.44	0.01841	2.6817068	Up
11	2‐[(1r,2r,5r)‐5‐Hydroxy‐2‐(3‐hydroxypropyl)cyclohexyl]‐5‐(2‐methyloctan‐2‐yl)phenol	1.54	0.00874	1.2145528	Up
12	Sphinganine	1.93	0.00015	1.3652839	Up
13	Isopropamide	2.39	0.00000	2.7823306	Up
14	4‐Propylphenol	1.76	0.00056	1.4833429	Up
15	Tyr‐Arg‐Leu	1.54	0.00245	1.5638980	Up
16	Oxoamide	1.35	0.00995	0.8603628	Down
17	1‐Methyluric acid	1.18	0.02722	0.7660336	Down
18	Gamma‐Caprolactone	1.66	0.00135	0.8895729	Down
19	Antipyrine	1.39	0.02303	0.8089353	Down
20	1‐Hexadecanoyl‐2‐octadecanoyl‐sn‐glycero‐3‐phosphocholine	2.17	0.00041	0.0159157	Down

### 3.4 Receiver Operating Characteristic (ROC) Curve Analysis

ROC analysis was performed to test biomarker potential, with the area under the curve (AUC) values derived from metabolomic profiles. ROC analysis was performed by comparing the HCE group with the healthy control group, determining the upper and lower limits of the measured values, the class intervals, and the optimal cutoff points. The cumulative frequency distribution was compiled according to the selected class interval, and sensitivity, specificity, and the false‐positive rate (1‐specificity) were calculated for all cutoff points. The AUC value reflects the diagnostic accuracy of the biomarkers. A larger AUC suggests a greater diagnostic value, with values ranging from 0.5 to 1.0. An AUC greater than 0.5 indicates some diagnostic utility, with values closer to 1.0 denoting superior performance. Specifically, AUC > 0.9 denotes strong diagnostic power; 0.7–0.9 reflects moderate accuracy; 0.5–0.7 indicates limited diagnostic utility, while an AUC of 0.5 suggests no discriminative ability.

In this study, 173 metabolites demonstrated an AUC greater than 0.9, indicating high discrimination power. To identify the most biologically relevant and practically valuable differential metabolites, a multi‐level screening strategy was applied: 1. All candidate metabolites were required to meet the criteria of *p* < 0.05 and VIP > 1 to ensure their significant contribution to classification and prediction. 2. Priority was given to metabolites involved in key biological pathways or directly related to the research objectives, supported by literature and database searches. 3. Focus was placed on metabolites with high FC values, emphasizing those exhibiting significant expression changes.

The AUC quantifies the ability of serum metabolites to differentiate HCE cases from controls. In the ROC plot, specificity is shown on the *x*‐axis and sensitivity on the *y*‐axis. The AUC value corresponds to the curve's enclosed area.

### 3.5 Optimization of Diagnostic Biomarkers

- Initially, 20 differential metabolites (VIP > 1, *p* < 0.05) were identified and tentatively proposed as candidate diagnostic biomarkers for HCE. However, to enhance clinical applicability and reduce model complexity, further screening was performed to identify a smaller, optimized panel with high diagnostic performance.
- Feature selection and ROC validation: A two‐step approach was employed for marker screening and optimization.

Cross‐validated recursive feature elimination (RFE): Using logistic regression combined with 10‐fold cross‐validation, we applied RFE to rank the metabolites based on their diagnostic relevance. We assessed the area under the ROC curve (AUC) for subsets of the top N features (*N* = 3 to 10). The best‐performing combination yielded an AUC of 0.94 (95% CI:0.88–0.99), with sensitivity and specificity of 85% and 89%, respectively. While the complete 20‐metabolite panel achieved a slightly higher AUC of 0.96, it showed signs of overfitting, with cross‐validated AUC dropping to 0.87.

To strike a balance between diagnostic accuracy and model robustness, we selected the top 5 differentially expressed metabolites based on their average AUC and minimal overfitting (standard deviation ± 0.03). These five metabolites achieved a combined AUC of 0.94 (95% CI: 0.88–0.99) and demonstrated strong diagnostic potential:

Isopropamide (VIP = 2.39, *p* < 0.001) was significantly upregulated (FC = 2.78), ranked first in RFE, with an individual AUC of 0.82;

Sphinganine (VIP = 1.93, *p* < 0.001) was upregulated 1.37‐fold (second in RFE, AUC = 0.81);

15(S)‐HETE‐d8 (VIP = 1.44, *p* = 0.018) had a 2.68‐fold increase in expression (third in RFE, AUC = 0.78);

Allantoic acid (VIP = 1.58, *p* = 0.008), the only significantly downregulated metabolite (FC = 0.64), ranked fourth in RFE; AUC = 0.75.

Tyr‐Arg‐Leu (VIP = 1.54, *p* = 0.002) was upregulated 1.56‐fold (fifth in RFE, AUC = 0.72).

**FIGURE 3:**
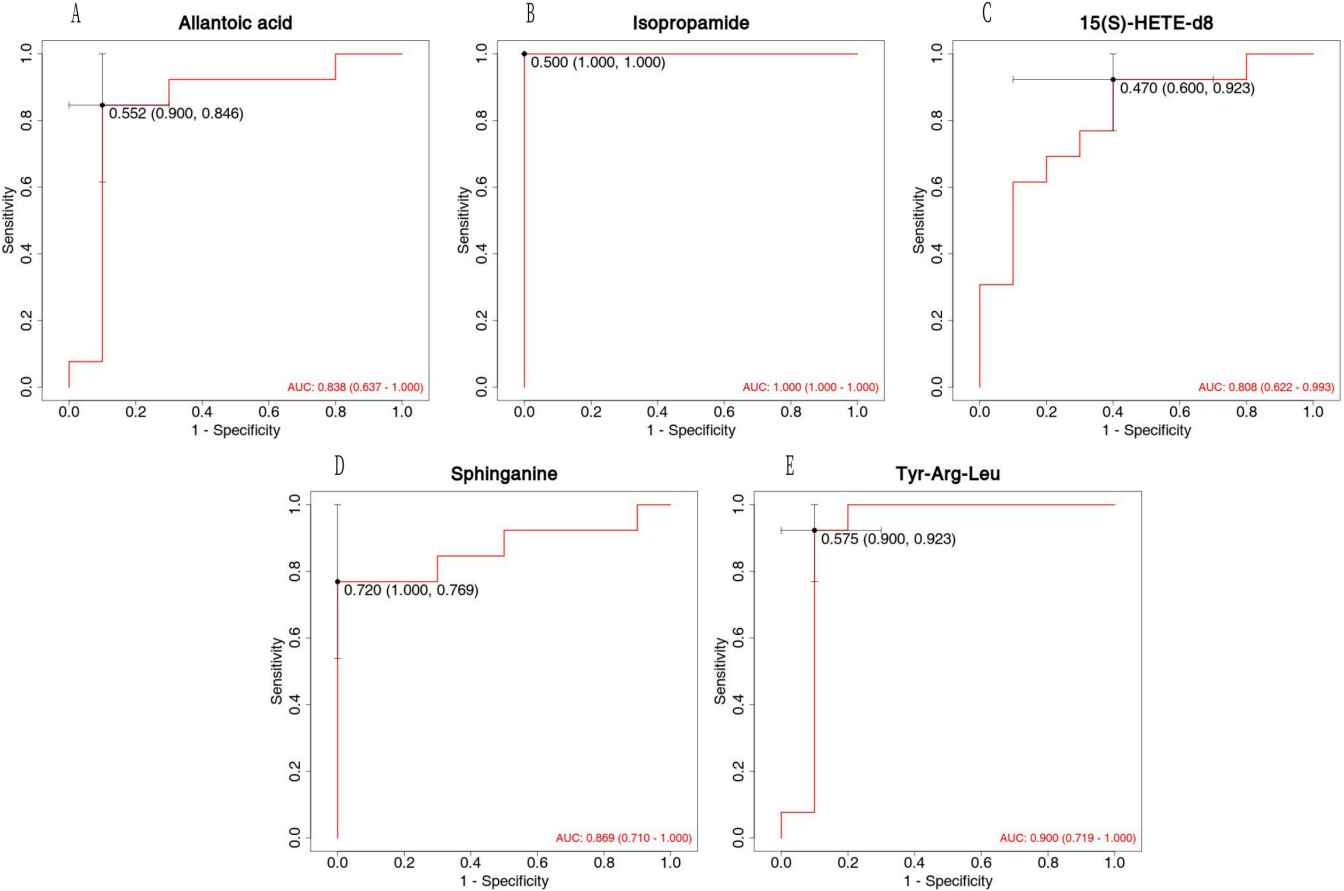
Analysis of serum differential metabolites and their correlation between HCE patients and healthy controls.

This optimized five‐metabolite panel provided a strong diagnostic signal, with superior classification performance compared to individual markers. The ROC curves and corresponding AUC values are presented in (Figure 3).

### 3.6 Clustering and Correlation Analysis of Differential Metabolites

#### 3.6.1 Clustering and Correlation Analysis

Following unit variance (UV) scaling of the metabolite data, a heatmap was generated using the ComplexHeatmap package in R. Hierarchical cluster analysis (HCA) was then applied to examine patterns of metabolite accumulation across the samples. UV‐scaled data were used for the clustering process, and an R script was employed to produce the heatmap.

**FIGURE 4:**
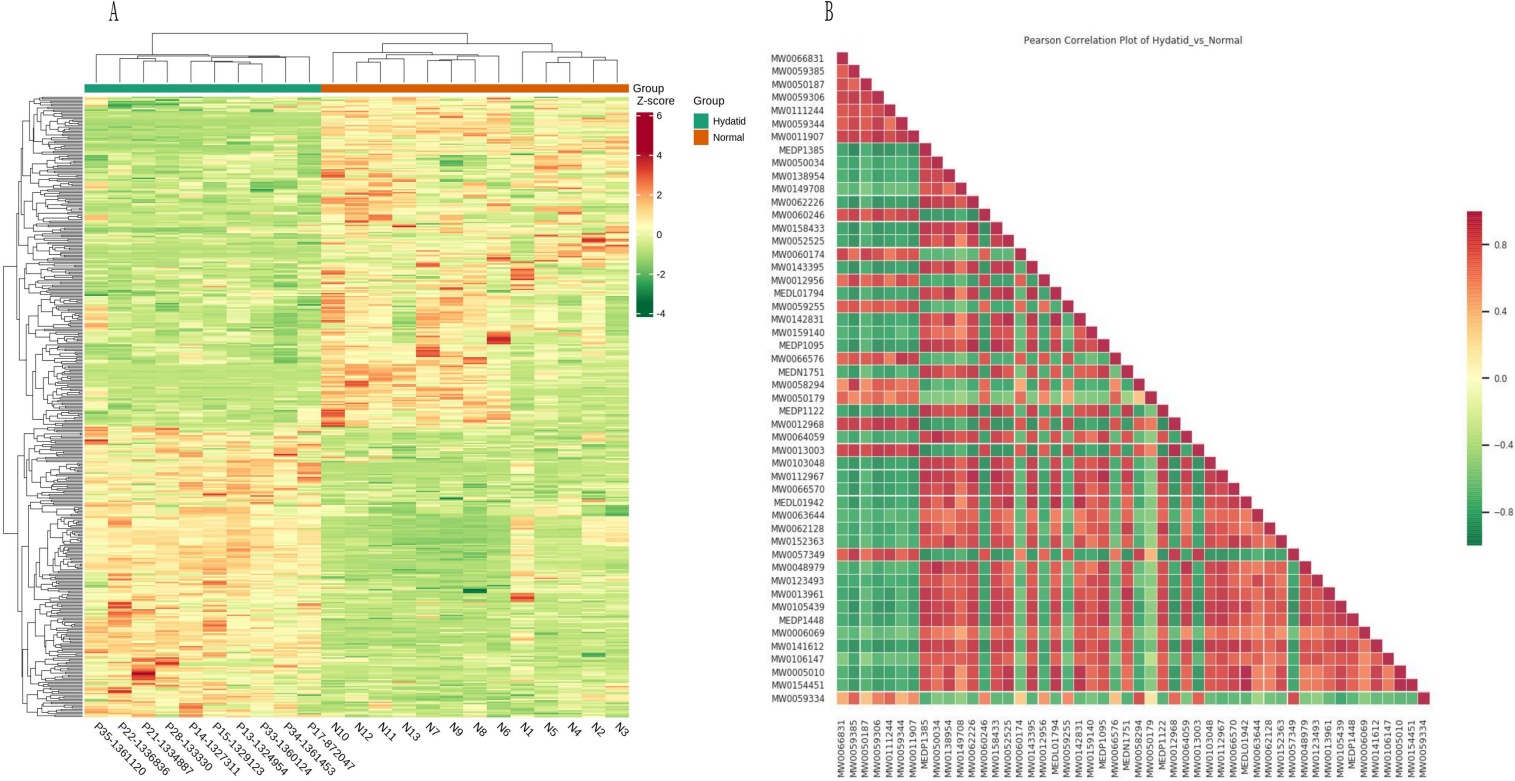
(A) Differential metabolites clustering heatmap. The horizontal axis displays sample information, while the vertical axis lists the differential metabolites. “Group” refers to the sample groupings. Colors represent normalized relative metabolite levels, with red indicating high levels and green indicating low levels. “Heatmap_col‐row_cluster” refers to the clustering of both columns and rows within the heatmap. (B) Correlation heatmap of differential metabolites: The horizontal and vertical axes display the names of the differential metabolites. Colors represent the Pearson correlation coefficients, with red indicating strong positive correlations and green indicating strong negative correlations. Deeper colors correspond to higher absolute coefficient values.

Hierarchical clustering using Ward's method grouped the differential metabolites into three distinct clusters, as shown in (Figure 4A). This clustering reveals distinct expression patterns of differentially expressed metabolites between the case and control groups. Biological replicates clustered together, indicating consistency and homogeneity within the replicates.

#### 3.6.2 Correlation Analysis of Differential Metabolites

After *Echinococcus granulosus* infestation, complex metabolic reactions and regulatory processes interact. Correlation analysis of metabolites provides insights into their proximity and interactions, helping to enhance our understanding of metabolic regulation during biological transitions. Pearson correlation analysis was applied to significant metabolites and QC samples. A higher correlation between QC samples (|r|closer to 1) suggests better stability and data quality of the test. The results of this analysis are presented below:

In this study, Pearson correlation analysis was performed on all differential metabolites, with data for the top 50 metabolites presented. The metabolites analyzed include glycerophospholipids, glycerides, fatty acids, amino acids, organic acids, terpenes, alcohols, amines, bile acids, aldehydes, ketones, esters, heterocyclic compounds, and hormone derivatives. Significant correlations between metabolites are shown in Figure 4 B (*p* < 0.05).

### 3.7 KEGG Classification and Enrichment Analysis of Differential Metabolites

#### 3.7.1 KEGG Classification

The identified metabolites map to 46 biological pathways spanning metabolism, cellular functions, organismal systems, disease processes, and signal transduction. Specifically, 53 metabolites were enriched in glycerophospholipid metabolism, 27 in arachidonic acid (AA) metabolism, 25 each in linoleic and alpha‐linolenic acid metabolism, 24 in glyceride metabolism, 5 in purine metabolism, and 4 in amino sugar and nucleotide sugar metabolism. A higher number of differential metabolites in a given pathway indicates more significant differences between the two groups for that pathway .

#### 3.7.2 KEGG Enrichment Analysis of Differential Metabolites

**FIGURE 5:**
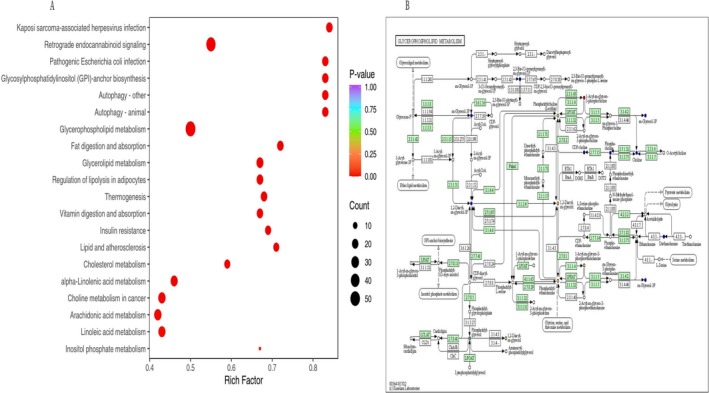
(A) KEGG enrichment of differential metabolites: Dots represent individual pathways. Redder colors indicate greater significance of enrichment (lower *p* values), while larger dots correspond to pathways with more differential metabolites. (B) KEGG pathway map of differential metabolites: Red indicates significantly upregulated metabolites in the experimental group, blue represents detected metabolites with no significant changes, green indicates significantly downregulated metabolites in the experimental group, and orange shows a combination of upregulated and downregulated metabolites.

KEGG online database was utilized for pathway enrichment analysis to identify the most relevant pathways and potential underlying mechanisms. Enrichment was assessed via the Rich Factor—the ratio of significant metabolites to total annotated compounds per pathway—where higher values indicate stronger association. Statistical significance was confirmed by *p* value, with smaller values denoting higher relevance. Dot size in Figure 5B represents the number of enriched metabolites per pathway. The 20 most significantly enriched pathways (lowest *p* values) are shown.

Key pathways in this study were selected in light of the KEGG enrichment analysis. Pathways with smaller *p* values indicate more significant enrichment. The top 20 pathways are presented in Figure 5A. Pathways with *p* < 0.05, such as glycerophospholipid metabolism, cholesterol metabolism, AA metabolism, and linoleic acid metabolism, were considered significantly enriched and potentially important for targeted therapeutic strategies.

The results suggest that lipid metabolism, autophagy, energy metabolism, signaling, and inflammation‐ and immune‐related pathways are the primary metabolic pathways enriched with differential metabolites. These findings underscore that *Echinococcus granulosus* infection may lead to metabolic disorders by disrupting lipid and energy metabolism, signaling pathways, and immune responses (Figure 5A).

#### 3.7.3 Cluster Analysis of KEGG Pathway Differential Metabolites

KEGG annotation data for differential metabolites were used to select the five most significantly enriched metabolic pathways for cluster analysis, with the goal of examining the variation patterns of these metabolites across different groups. Pathways with fewer than five differential metabolites were excluded from the analysis (Figure 5A).

### 3.8 Key Metabolic Pathway Analysis

KEGG analysis revealed several significantly enriched pathways, with glycerophospholipid metabolism identified as the most critical. When a metabolic pathway is disrupted, related metabolites often exhibit abnormal levels and show significant enrichment in that pathway. These metabolic alterations could provide valuable insights into the pathogenesis of HCE.

### 3.9 Functional Annotation of Differential Metabolites

Metabolites interact within the body to form various metabolic pathways. KEGG database annotation was utilized to categorize the differential metabolites, displaying only the pathways that include these metabolites.

As shown in Figure 5B, glycerophospholipid metabolism is a key pathway. In the experimental group, *1‐acyl‐SN‐glycero‐3‐phosphocholine* is significantly upregulated, while *phosphatidylglycerol* is significantly downregulated. Metabolites such as phosphatidylcholine, 1,2‐diacyl‐sn‐glycerol, 1‐acyl‐sn‐glycero‐3‐phosphoethanolamine, and phosphatidylethanolamine exhibit both upregulation and downregulation.

The differential metabolites within this key pathway, along with the common enzymes and receptor proteins involved in their synthesis, as well as their specific biological significance, will require further validation through subsequent research.

## 4 Discussion

It is well established that certain diseases, in order to adapt to the human body's environment, may induce metabolic reprogramming in patients. This reprogramming involves the alteration of metabolic pathways, shifting the direction and abundance of metabolites within these pathways. These changes create favorable conditions for disease progression by providing essential substrates and increasing energy supply, thereby accelerating the disease process (Zhou et al. 2019). As a new focus of omics research, following genomics, transcriptomics, and proteomics, metabolomics uses techniques such as nuclear magnetic resonance (NMR) and MS to qualitatively and quantitatively analyze hundreds to thousands of biochemical intermediates and end‐products (e.g., amino acids, lipids, and organic acids) in organisms. This approach offers detailed insights into the characteristics of endogenous metabolites and their responses to both internal and external changes, with wide‐ranging applications in disease diagnosis, drug development, and the study of biological functions (Hang and Shen 2021).

In this study, a total of 2030 metabolites were detected, of which 20 showed significant differential abundance and were initially associated with HCE. To enhance clinical applicability and minimize redundancy, we refined this panel using RFE, ultimately identifying five key biomarkers—isopropamide, sphinganine, 15(S)‐HETE‐d8, allantoic acid, and Tyr‐Arg‐Leu. This optimized combination achieved high diagnostic accuracy, with an AUC of 0.94, indicating strong discriminatory power.

However, the small sample size in this study may result in false‐positive findings, particularly among high‐AUC metabolites. Although cross‐validation and permutation testing supported the robustness of several biomarkers (e.g., isopropamide and sphinganine), further validation in large, multi‐center cohorts is essential before clinical translation. Previous studies have reported potential overestimation of AUC values in small‐sample metabolomics research (Lin 2018).

Previous research has shown that HCE lesions are characterized by a highly immunosuppressive microenvironment, including macrophage subset imbalance, Th2 polarization of CD4^+^ T cells, and marked infiltration of regulatory T cells (Tregs), all contributing to parasite immune escape (Wang et al. 2023). In this study, the differential metabolite sphinganine was significantly upregulated and may participate in host immune regulation or parasitic escape mechanisms. Supporting this, animal models and patient samples have demonstrated upregulation of M2 macrophage‐related genes (Arg1, Ym1) and cytokines (IL‐10, TGF‐β) at lesion sites, with their mRNA levels positively correlated with cyst diameter and growth rate (De Biase et al. 2023).

Parasitic infections such as *Schistosoma mansoni* have been shown to reprogram host cell metabolism in the liver and intestine, facilitating nutrient uptake to support parasite development. Eggs store resources like neutral lipids—critical for forming phospholipid‐rich membranes during miracidia development—and may utilize their sieve‐like eggshell pores to extract lipids from host tissues. This metabolic manipulation likely contributes to host lipid and glycogen depletion during infection (von Bülow et al. 2023). In this study, serum levels of the differential metabolite N‐[(3S)‐tetrahydro‐2‐oxo‐3‐furanyl] hexadecanamide were elevated in HCE patients versus healthy controls. This lipid‐like metabolite may interfere with host lipid metabolism, suppress immunity, or provide nutritional support to the parasite, potentially contributing to HCE pathogenesis. In contrast, serum Dacarbazine levels were reduced in HCE patients, possibly indicating impaired hepatic metabolic enzyme function and altered drug clearance capacity.

The laminated cyst wall of *Echinococcus granulosus* is primarily composed of host‐derived phospholipids. To facilitate its membrane biosynthesis, the parasite secretes phospholipase A2 (PLA2), which hydrolyzes host cell membrane phospholipids to release free fatty acids (e.g., arachidonic acid) and lysophospholipids (Hervé et al. 2023). These metabolites are crucial building blocks for the parasite's own membrane system. In addition, by interacting with the host's S1PR1 receptor, the parasite can inhibit NLRP3 inflammasome activation and reduce IL‐1β secretion (Wang et al. 2017). Lyso phosphatidylcholine (LPC) further inhibits macrophage‐mediated inflammation, contributing to immune evasion (Liu et al. 2024). This strategy likely reflects a broader reprogramming of host lipid metabolism, including excessive consumption of adipocyte membrane components. In this study, we observed significant downregulation of 1‐hexadecanoyl‐2‐octadecanoyl‐sn‐glycero‐3‐phosphocholine (a phosphatidylcholine).

Parallels can be drawn from *Toxoplasma gondii*, which exploits host lipid droplets as mobilizable reservoirs of neutral lipids and fatty acids. Infected cells display an accumulation of lipid droplets, which the parasite utilizes by absorbing oleic acid and storing excess lipids as cytoplasmic triacylglycerides. Transient endocytic structures form on the parasite membrane in response to lipid abundance and are implicated in both lipid and host protein uptake (Dou et al. 2014).

Similarly, *Echinococcus granulosus* expresses low‐density lipoprotein receptor (LDLR)‐like proteins to directly take up cholesterol from the host plasma, further suppressing the inflammatory response (Fan et al. 2024). Parasite‐derived prostaglandins shift the immune response toward a Th2 phenotype. In this study, the differential metabolite 15(S)‐HETE‐d8 was significantly upregulated in the HCE group. As a downstream metabolite of arachidonic acid, it may reflect the host's lipid‐mediated inflammatory response. By activating the PPARγ pathway, 15(S)‐HETE‐d8 inhibits fatty acid oxidation in host hepatocytes and promotes a metabolic shift toward glycolysis, thereby increasing lactate production (Xu et al. 2021).

Additionally, in this study, prostaglandin A1 ethyl ester was significantly upregulated in HCE patient serum. Prostaglandins (such as PGA1) are known to inhibit the expression of MHC‐II in dendritic cells, impair CD4^+^ T cell activation, and promote a Th2‐type immune response (e.g., increased IL‐4/IL‐10), all of which support parasite persistence (Sharma et al. 2018). The significant upregulation of prostaglandin A1 ethyl ester in this study may also contribute to membrane stabilization, apoptosis regulation, and parasite signal transduction.

This study identified 46 metabolic pathways, eight of which are key pathways playing a crucial role in the disease development process. Below, we discuss the biological functions of these pathways and their potential contributions to the pathogenesis of HCE. The 20 differentially expressed metabolites identified in this study are closely related to several metabolic pathways, including glycerophospholipid metabolism, cholesterol metabolism, arachidonic acid metabolism, linoleic acid metabolism, autophagy, energy metabolism, retrograde endocannabinoid signaling, and pathways related to inflammatory and immune responses.

1 Glycerophospholipid metabolism pathway: This lipid metabolism pathway is central to cell membrane structure and inflammation. It regulates the phospholipid composition of cell membranes, which influences signal transduction and inflammation. *Echinococcosis* infection may disrupt host cell membrane stability, leading to pathway abnormalities (O'Donnell et al. 2019).

Studies suggest that following *echinococcosis* infection, the host liver creates an “immunosuppressive microenvironment.” Glycerophospholipid metabolism may influence antiparasitic immunity by modulating the activity of immune cells, including T cells and NK cells (Dejong et al. 2007). Research from the Shanghai Institute of Nutrition and Health, Chinese Academy of Sciences, has shown that elevated specific glycerophospholipids, such as phosphatidylcholine, can increase the risk of metabolic diseases (Chen et al. 2022). This suggests that *echinococcosis* infection might similarly affect the host's metabolic health (Trostchansky et al. 2021).

2 Cholesterol metabolism pathway: This pathway is directly linked to lipid metabolic disorders. Cholesterol synthesis, transport, and metabolism are integral to lipid homeostasis and immune regulation. Parasites may hijack host cholesterol for their survival, leading to metabolic imbalances (Vallochi et al. 2018).

Studies show that cholesterol metabolites, such as oxidized cholesterol, can modulate the host's immune response to *echinococcosis* by affecting immune cell function (Abu‐Omar et al. 2005). The role of cholesterol metabolism in liver diseases, such as fatty liver and liver cancer, has been well documented. For example, the accumulation of oxidized cholesterol can cause hepatic inflammation and fibrosis, similar to the pathological changes observed in *echinococcosis* (Das 2021).

3 AA metabolism pathway: The AA metabolism pathway is involved in inflammatory responses and immune regulation during *echinococcosis* infection. AA is metabolized through COX, LOX, and CYP450 pathways to produce inflammatory mediators like PGE2 and leukotrienes (LTs), which can exacerbate inflammation and tissue damage in hepatic echinococcosis (Genchi 2017). Additionally, PGE2 and other AA metabolites may promote parasite immune evasion by modulating immune cell function and altering the host's immune response to infection (McAllan et al. 2013).
4 Linoleic acid metabolism pathway: As an essential fatty acid metabolism pathway, linoleic acid metabolism may reflect the host's metabolic adaptive changes, affecting energy supply and cell membrane integrity (Lin et al. 2019). It may also indicate metabolic adaptation to infection or nutritional competition. Previous evidence has documented that elevated COX2 expression is associated with various inflammatory diseases. For instance, PGE2 can suppress immune cell activity through EP2/EP4 receptor signaling, which may offer insights into the immunosuppressive mechanisms in hepatic echinococcosis (Zhang et al. 2021).
5 Autophagy pathway: The autophagy‐related pathway is crucial in the immune response and cell metabolism in *echinococcosis*. Research on hepatic echinococcosis has shown that autophagy is associated with disease progression, with altered expression of autophagy‐related proteins. This suggests that the autophagy pathway may be crucial in the pathogenesis of the disease (Chen et al. 2021).
6 Energy metabolism pathways (thermogenesis and insulin resistance): In chronic HCE infection, immune evasion occurs. Its mechanism may be explored by detecting changes in immunocyte energy metabolism (Song et al. 2017). In this study, the upregulation of the sugar derivative (D‐Erythrulose 4‐phosphate) may indicate enhanced sugar metabolism to meet the energy needs of either the host or parasite. Energy metabolic disorders may be implicated in *echinococcosis* and are likely to play a significant role in HCE pathogenesis. Parasites adapt their energy metabolism to survive within the host environment and evade immune attacks, while changes in host energy metabolism influence disease progression (Gao and Zhang 2013). For example, a study by Guo et al. evaluated the efficacy of dual‐regulated energy metabolism nanoparticles (Atovaquone‐Albendazole nanoparticles) against HCE and found that Atovaquone combined with Albendazole enhanced anti‐parasite effects, likely by regulating the parasite's energy metabolism (Lu et al. 2013).
7 Retrograde endocannabinoid signaling: This pathway suggests that signaling mechanisms may be pivotal in metabolic changes associated with *echinococcosis*. Studies have shown that the MAPK pathway is critical in the pathogenesis of HCE (Chong et al. 2022). By modulating intracellular signaling, the MAPK pathway affects cell proliferation, differentiation, and apoptosis. Abnormal activation of this pathway in HCE may be linked to parasite growth and development (Zhang et al. 2025). In this study, allantoic acid, a purine metabolite, was significantly downregulated. Serum levels of purine metabolites in HCE patients were lower than in healthy volunteers, indicating potential abnormalities in purine metabolism. Such disruptions may affect nucleotide metabolism, as nucleotides are crucial for DNA and RNA synthesis, biosynthetic processes, and overall metabolic regulation (Wei et al. 2018).
8 Inflammatory and immune response pathways: Inflammatory and immune signaling play central roles in HCE pathogenesis. Notably, NF‐κB activation drives the release of pro‐inflammatory mediators, exacerbating local immune responses. Persistent NF‐κB dysregulation has been associated with the chronic inflammation characteristic of HCE (Lu and Zhou 2024).

In addition, amino acid metabolism pathways, which are essential for protein synthesis and breakdown, significantly influence the growth and reproduction of HCE. The liver, being the primary organ affected by echinococcosis, is exploited for nutrients by the parasitic cysts. As these cysts expand, they induce structural and functional changes in the liver (Ezquerro et al. 2020; Gong et al. 2024). Previous studies have shown that phenylalanine is converted to tyrosine exclusively in the liver (McManus et al. 2012; Yasen et al. 2021). As amino acid catabolism predominantly occurs in the liver, hepatic dysfunction can result in decreased levels of free branched‐chain amino acids and elevated free amino acids, such as phenylalanine, tryptophan, and tyrosine (Eckert and Deplazes 2004; Tietge et al. 2003).

Recent studies have documented that hepatic echinococcosis can profoundly alter amino acid metabolism, and circulating amino acid levels reflect the metabolic balance between muscle tissue and the liver (Cheng et al. 2020). In this study, the differential metabolite Tyr‐Arg‐Leu was significantly upregulated. As a tyrosine‐containing residue, it may contribute to antioxidant activities. Its upregulation in the serum of HCE patients suggests potential liver damage and disruption of tyrosine metabolism during *Echinococcus granulosus* infection. However, further experiments are needed to clarify the underlying mechanisms.

LC‐MS/MS is an effective tool for metabolomic studies of hydatid disease. It offers high repeatability, the ability to detect a wide range of metabolites simultaneously, and rapid analysis. This approach enables characterization of metabolic disruptions triggered by *Echinococcus granulosus* through small‐molecule profiling. Furthermore, combining imaging techniques with metabolomics could aid in the early diagnosis of hydatid disease (Cai et al. 2012; Van Zijl et al. 2018).

Limitations of this study: This preliminary study investigating metabolomic differences between HCE patients and healthy people has several limitations, mainly due to the small sample size: (1) Small Sample Size: With only 10 HCE patients and 13 controls, the statistical power is limited, and the generalizability of the results may be affected. (2) Population Heterogeneity: The small cohort may not adequately represent population diversity. Confounding variables such as age, sex, disease stage, and comorbidities may not be evenly distributed. (3) Overfitting Risk: Due to the high dimensionality of metabolomics data, small sample sizes increase the likelihood of overfitting. Although cross‐validation or strict statistical corrections was used to reduce this risk, it remains a concern. Despite these limitations, our data provide valuable insights and potential biomarker candidates for further investigation. In future work, we aim to expand the sample size to at least 30 participants per group and establish an independent validation cohort to verify our findings and enhance the reliability and accuracy of the study.

Even with a limited number of biological samples, they allow for the verification of multiple markers based on prior differential proteomics and metabolomics data (Zhang and Li 2022). The integration with other omics technologies (e.g., genomics and proteomics) could provide additional support for the experimental results.

## 5 Conclusion

In this study, a metabolomics approach based on LC‐MS/MS was utilized to examine the metabolic alterations associated with HCE. A total of 20 potential differential metabolites were identified, primarily involving 8 key metabolic pathways, with lipid metabolism emerging as the most significantly affected. The findings suggest that inflammatory and immune responses may play significant roles in the pathogenesis of echinococcosis. The identified candidate biomarkers offer novel insights into the molecular mechanisms underlying HCE and may serve as promising indicators for early diagnosis and potential targets for therapeutic intervention.

## Conflicts of Interest

The authors declare no conflicts of interest.

## Data Availability

All data supporting the findings of this study are included within the article.
